# EZH2-mediated inhibition of microRNA-22 promotes differentiation of hair follicle stem cells by elevating STK40 expression

**DOI:** 10.18632/aging.103165

**Published:** 2020-07-12

**Authors:** Bingjie Cai, Min Li, Yunpeng Zheng, Yakun Yin, Fangcao Jin, Xuyang Li, Juan Dong, Xiaoyan Jiao, Xiaojun Liu, Kun Zhang, Dongqin Li, Junmin Wang, Guangwen Yin

**Affiliations:** 1 Department of Dermatology, The First Affiliated Hospital of Zhengzhou University, Zhengzhou 450052, P. R. China; 2 Department of Dermatology, Henan Provincial People's Hospital, Zhengzhou 450003, P. R. China; 3 Henan Province Medical Instrument Testing Institute, Zhengzhou 450018, P.R. China; 4 School of Life Sciences, Zhengzhou University, Zhengzhou 450001, P.R. China; 5 Department of Anatomy, College of Basic Medical Sciences, Zhengzhou University, Zhengzhou 450000, P.R. China

**Keywords:** hair growth, EZH2, microRNA-22, STK40, hair follicle stem cell differentiation

## Abstract

Hair follicle stem cells (HFSCs) contribute to the regeneration of hair follicles (HFs), thus accelerating hair growth. microRNAs (miRs) are potential regulators in various cellular processes, including HFSC proliferation and differentiation. This study proposed a potential target, enhancer of zeste homolog 2 (EZH2) for facilitating hair growth, due to its function over HFSC activities by mediating the miR-22/serine/threonine kinase 40 (STK40)/myocyte enhancer factor 2 (MEF2)/alkaline phosphatase (ALP) axis. Gain- and loss-of-function approaches were adopted to explore the roles of EZH2, miR-22, and STK40 in the proliferation and apoptosis of HFSCs, along with the functional relevance of MEF2-ALP activity. STK40 was elevated during HFSC differentiation, which was found to facilitate HFSC proliferation, but impede their apoptosis by activating MEF2-ALP. Mechanically, miR-22 targeted and inversely regulated STK40, which inhibited MEF2-ALP activity to impede HFSC proliferation and differentiation. Moreover, EZH2 elevated the STK40 expression by repressing miR-22 to promote the proliferation and differentiation of HFSCs. Furthermore, *in vivo* experiments further validated the roles of EZH2 and STK40 on hair follicle neogenesis and hair growth. Collectively, EZH2 elevated the STK40 expression by downregulating miR-22, consequently accelerating differentiation of HFSCs and hair growth, which sheds light on the underlying molecular mechanism responsible for hair growth.

## INTRODUCTION

Hair follicle (HF) is characterized by the ability to regenerate numerous times from cycles of growth (anagen), regression (catagen) to rest (telogen) in adult life [1]. Specifically, the modulation and cycling of HF growth are highly conserved and associated with several epithelial-mesenchymal interactions, which are fundamental for the formation of epidermal appendages [2]. Hair follicle stem cells (HFSCs) can radically sustain the self-renewal of skin tissues, and the cells exhibiting the transition ability from HFSCs into differentiated epidermal cells are known as the transit-amplifying (TA) cells [3]. The progeny of stem cells *via* the TA phase shows rapid cell division cycles before differentiating [4]. Elucidating mechanisms responsible for mediating the differentiation of HFSCs is therefore critical for inducing extensive HF neogenesis and hair growth.

Of note, microRNAs (miRs) have demonstrated critical function in hair cycle-associated tissue remodeling and HF development [5]. miR-22 overexpression was revealed to facilitate hair loss due to repressed hair keratinocyte differentiation and keratinocyte progenitor expansion [6]. Interestingly, miR-22 was reported to be repressed by enhancer of zeste homolog 2 (EZH2) in hepatocellular carcinoma [7]. EZH2 essentially functioned as a histone methyltransferase to mediate gene expression as a catalyst of the polycomb repressive complex 2 [8]. EZH2 has been documented to serve as a modulator of HFSC proliferation and differentiation [9]. In addition, bioinformatics analysis predicted serine/threonine kinase 40 (STK40) as a potential downstream target of miR-22. STK40 suppression was synchronous with a lower expression of hair differentiation markers and reduced hair growth [10]. STK40 served as a new favorable regulator of skeletal myoblast differentiation and fetal skeletal muscle formation impaired fetal skeletal muscle formation and maintained the transcriptional activities of myocyte enhancer factor 2 (MEF2) [11]. As a pleiotropic transcription factor, MEF2 was regarded as an essential regulator in the development of muscles [12], and could enhance the activity of alkaline phosphatase (ALP) [13], a dermal papilla marker [14]. The aforementioned findings provided a possible mechanism underlying the involvement of EZH2, miR-22 and STK40-dependent MEF2-ALP axis in HFSC differentiation and hair growth. Thus, we established different mouse models to explore the underlying regulatory network. A comprehensive understanding of the molecular regulation of HFSC differentiation could provide an insight on altering the process of hair growth.

## RESULTS

### STK40 expression is elevated during HFSC differentiation into TA cells

**Figure 1:**
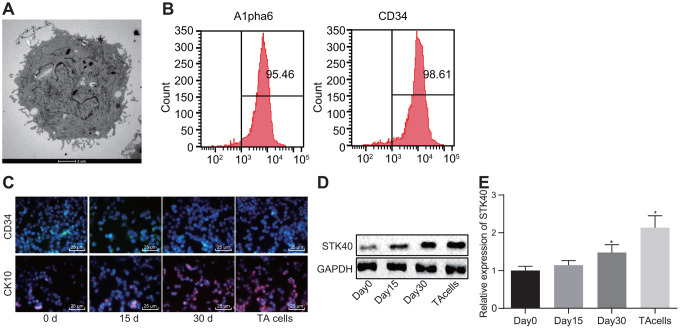
**STK40 is highly expressed during HFSC differentiation into TA cells.** (**A**) The growth of HFSCs observed under a microscope (5000 ×). (**B**) The expression of Alpha6 and CD34 determined by flow cytometry to sort HFSCs. (**C**) HFSC differentiation markers queried using immunofluorescence assay (400 ×). (**D**) Protein expression of STK40 normalized to GAPDH during HFSC differentiation determined using Western blot analysis. (**E**) Relative expression of STK40 during HFSC differentiation determined using RT-qPCR. * *p* < 0.05 *vs.* day 0; Measurement data were expressed as mean ± standard deviation. One-way ANOVA was utilized to compare data among multiple groups, followed by Tukey’s post hoc test. Cell experiments were conducted in triplicates.

A prior study demonstrated the vitality of STK40 in keratinocyte growth and hair differentiation by functioning as a regulator of the expression of significant hair follicle program regulators [10]. To understand the role of STK40 in HFSC differentiation, we isolated HFSCs from WT mice (Figure 1A). Then, 6 days after culturing, HFSCs exhibited growth (Figure 1A). Eight days later, HFSCs were in their exponential growth phase (Figure 1A). Flow cytometry (Figure 1B) was employed to sort and characterize HFSCs based on evaluation of the expression of Alpha6 and CD34, while immunofluorescence (Figure 1C) was adopted to detect the expression of the HFSC differentiation markers, and the results were indicative of successful differentiation of HFSCs into TA cells. As shown in Figure 1D, 1E, the expression of STK40 was determined using Western blot analysis and Reverse transcription quantitative polymerase chain reaction (RT-qPCR) during HFSC differentiation, and the results of which showed a moderate rise in STK40 expression after 15 days, remarkable increase was evident 30 days later, and its expression peaked in the final stage of TA cells. Conjointly, the expression of STK40 was up-regulated during HFSC proliferation and differentiation.

### STK40 promotes proliferation and differentiation of HFSCs *via* MEF2-ALP axis

**Figure 2:**
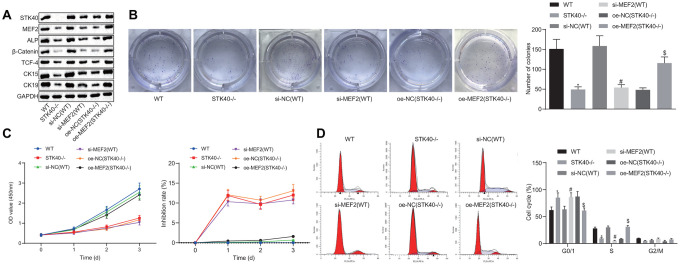
**STK40 expedites the proliferation and differentiation of HFSCs *via* MEF2-ALP axis.** (**A**) Expression of STK40, MEF2, ALP, differentiation-related proteins (β-catenin, TCF-4), and TA cell differentiation markers (CK15, CK19) normalized to GAPDH determined by Western blot analysis. (**B**) Colony forming capacity of HFSCs determined by colony formation assay. (**C**) HFSC proliferation and viability evaluated by MTS assay. (**D**) HFSC cell cycle changes assessed by flow cytometry. * *p* < 0.05 *vs.* WT mice; # *p* < 0.05 *vs.* si-NC (WT) group; $ *p* < 0.05 *vs.* oe-NC (STK40^-/-^) group; Measurement data were expressed as mean ± standard deviation. One-way ANOVA was utilized to compare data among multiple groups, followed by Tukey’s post hoc test. Cell experiments were conducted in triplicates.

For a better understanding of the regulatory role of STK40 on HFSC proliferation and differentiation, we extracted HFSCs from the STK40^-/-^ mice. A prior study highlighted the ability of STK40 to enhance the transcriptional activity of MEF2 and promote its expression [11]. MEF2 can further upregulate the expression of ALP, a dermal papilla marker [13, 14]. Thus, we hypothesized that STK40 facilitated HFSC differentiation *via* the MEF2-ALP axis. To elucidate this hypothesis, we conducted Western blot analysis to determine the expression of HFSC differentiation-related proteins (β-catenin, TCF-4), and TA cell differentiation markers (CK15, CK19). As depicted in Figure 2A, the results demonstrated that protein expression of STK40, MEF2, ALP, β-catenin, TCF-4, CK15 and CK19 was markedly reduced in response to treatment with si-MEF2 (STK40^-/-^), while the effect of STK40^-/-^ was abrogated upon treatment with oe-MEF2 (STK40^-/-^) (*p* < 0.05). The results obtained from the colony formation and 3-(4,5-dimethylthiazol-2-yl)-5-(3-carboxymethoxyphenyl)-2-(4-sulfopheny l)-2H-tetrazolium, inner salt (MTS) assays (Figure 2B, 2C) revealed markedly reduced colony formation and proliferation ability in the STK40^-/-^ and si-MEF2 (WT) groups, while the inhibition rates in these two groups was increased. The effect of STK40 knockout on colony formation and proliferation was abrogated upon treatment with oe-MEF2 (STK40^-/-^) (*p* < 0.05). Flow cytometry (Figure 2D) revealed that more HFSCs were arrested in the G0/G1 phase in response to MEF2 silencing or STK40 knockout, while this effect of STK40 knockout could be reversed by the delivery of oe-MEF2 (*p* < 0.05). Collectively, STK40 facilitated the proliferation and differentiation of HFSCs *via* MEF2-ALP axis.

### STK40 overexpression promotes HF keratinocyte differentiation and hair growth, but inhibits apoptosis *in vivo*

**Figure 3:**
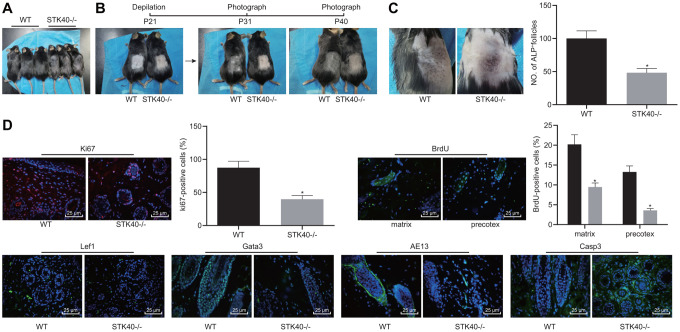
**STK40 knockout inhibits HF keratinocyte differentiation and hair growth, but facilitated the apoptosis.** (**A**) Hair loss conditions of STK40^-/-^ mice at 30 days postnatal. (**B**) Delayed hair growth in STK40^-/-^ mice. (**C**) HF neogenesis in STK40^-/-^ mice and WT mice as determined by ALP staining. (**D**) Expression of the corresponding proliferation, differentiation, and apoptosis markers in the skin samples of STK40^-/-^ and WT mice as detected by immunofluorescence assay (400 ×). * *p* < 0.05 *vs.* WT mice; Measurement data were expressed as mean ± standard deviation. Unpaired *t* test was adopted to analyze the differences between two experimental groups. n = 15.

We found that HFSC proliferation and differentiation were impeded in STK40^-/-^ mice. For a better analysis of the mechanism underlying STK40 regulating hair growth, we firstly photographed WT and STK40^-/-^ mice to assess the hair growth. As shown in Figure 3A, STK40^-/-^ mice exhibited obvious hair loss. Hair regeneration was assessed in the excisional wounds inflicted on the back, and the results indicated remarkably delayed hair growth in the STK40^-/-^ mice (Figure 3B). ALP staining (Figure 3C) was conducted to investigate the degree of NF neogenesis, which revealed a notable lower number of HFs in STK40^-/-^ mice than WT mice (*p* < 0.05). Additionally, immunofluorescence assay results (Figure 3D) demonstrated markedly reduced numbers of cells positive for Ki67, BrdU (in the matrix and the prehair cortex), Lef1 and Gata-3 in the skin samples from STK40^-/-^ mice, suggesting hindered HFSC proliferation, migration and keratinocyte differentiation in STK40^-/-^ mice. In the STK40^-/-^ mice, the expression of the hair cortex marker AE13 was also affected, which was positive in WT mice, suggesting impaired hair cortex formation. In addition, markedly increased expression of cleaved caspase 3 in HFs and reduced apoptosis of HF keratinocyte were observed in the STK40^-/-^ mice (*p* < 0.05). The aforementioned results suggested that STK40 facilitated the differentiation of HF keratinocytes and hair growth, but consequently repressed their apoptosis.

### miR-22 inversely regulates STK40 expression to inhibit MEF2-ALP activity

**Figure 4:**
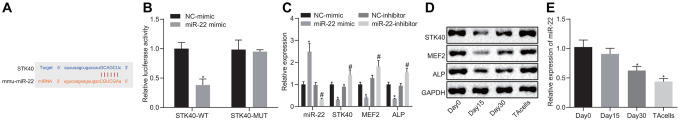
**miR-22 inhibits MEF2-ALP activity by targeting STK40.** (**A**) Bioinformatics prediction of the binding sites between miR-22 and STK40. (**B**) Dual-luciferase reporter gene assay showing the binding between miR-22 and STK40; * *p* < 0.05 *vs.* WT NC group. (**C**) mRNA expression of STK40, MEF2 and ALP and miR-22 expression in HFSCs in response to transfection with the miR-22 mimic, NC mimic, miR-22-inhibitor or NC-inhibitor determined by RT-qPCR. * *p* < 0.05 *vs.* NC-mimic; # *p* < 0.05 *vs.* NC-inhibitor. (**D**) STK40, MEF2 and ALP protein expression in HFSCs normalized to GAPDH in response to transfection with miR-22 mimic, NC mimic, miR-22-inhibitor or NC-inhibitor determined by Western blot assay. (**E**) The expression of miR-22 during HFSC differentiation determined by RT-qPCR; * *p* < 0.05 *vs.* day 0. Measurement data were expressed as mean ± standard deviation. Unpaired *t* test was adopted to analyze the differences between two experimental groups, while one-way ANOVA was utilized to compare data among multiple groups, followed by Tukey’s post hoc test. Cell experiments were conducted 3 times independently.

To study the upstream regulatory mechanism of STK40, we adopted bioinformatics analysis (Figure 4A) to predict the potential binding sites between miR-22 and STK40. Previous evidence has demonstrated an association between miR-22 and hair growth [6]. Dual-luciferase reporter gene assay (Figure 4B) was adopted to confirm the binding relationship between STK40 and miR-22. Results demonstrated diminished luciferase activity in the miR-22 mimic + WT-STK40 co-transfection group (*p* < 0.05), but no difference was observed in the mutated (MUT) 3’UTR group (*p* > 0.05). In addition, RT-qPCR (Figure 4C) results also revealed a marked decline in the mRNA expression of STK40, MEF2 and ALP, along with an elevation in the miR-22 expression upon transfection with miR-22 mimic, but conflicting changes were observed in response to miR-22-inhibitor transfection (*p* < 0.05). Western blot analysis (Figure 4D) results identified a marked reduction in the protein expression of STK40, MEF2 and ALP in response to miR-22 mimic transfection, but an opposite increase was seen in response to miR-22-inhibitor transfection (*p* < 0.05). The expression of miR-22 during HFSC proliferation and differentiation was determined by RT-qPCR (Figure 4E), which revealed that miR-22 expression exhibited a moderate descent 15 days later, dramatically diminished 30 days later, and exhibited the lowest expression in TA cells. Conjointly, miR-22 targeted STK40 and down-regulated its expression, thereby inhibiting the MEF2-ALP pathway.

### miR-22 attenuates HFSC proliferation and differentiation by repressing STK40 and MEF2-ALP activity

**Figure 5:**
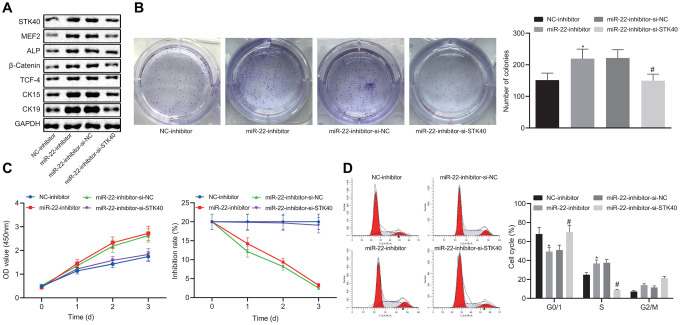
**miR-22 inhibits proliferation and differentiation of HFSCs by downregulating STK40 and suppressing MEF2-ALP activity.** (**A**) Protein expression of STK40, MEF2, ALP, differentiation-related proteins (β-catenin, TCF-4), and TA cell differentiation markers (CK15, CK19) in HFSCs normalized to GAPDH determined by Western blot analysis. (**B**) Colony forming capacity of HFSCs determined by colony formation assay. (**C**) HFSC proliferation and viability evaluated by MTS assay. (**D**) HFSC cell cycle changes revealed by flow cytometry. * *p* < 0.05 *vs.* NC-inhibitor group; # *p* < 0.05 *vs.* miR-22-inhibitor + si-NC group; Measurement data were expressed as mean ± standard deviation. One-way ANOVA was utilized to compare data among multiple groups, followed by Tukey’s post hoc test. Repeated measures ANOVA was adopted to analyze data among multiple groups at different time points, followed by Bonferroni posttest. Cell experiments were conducted 3 times independently.

To further elucidate the regulatory role of miR-22-STK40 axis on HFSC proliferation and differentiation, we extracted HFSCs from WT mice, followed by transfection with different plasmids. Western blot analysis results shown in Figure 5A revealed increased protein expression of STK40, MEF2, ALP, β-catenin, TCF-4, CK15 and CK19 upon transfection with the miR-22-inhibitor, while this effect of miR-22-inhibitor was reversed in response to co-transfection with si-STK40 (*p* < 0.05). Colony formation assay and MTS results (Figure 5B, 5C) revealed markedly improved colony forming and proliferation abilities, with evidently reduced inhibition rates of HFSCs in response to miR-22-inhibitor transfection, while the effect of miR-22-inhibitor was abrogated upon co-transfection with si-STK40 (*p* < 0.05). Flow cytometry (Figure 5D) results revealed that the number of HFSCs in the G0/G1 phase was reduced in response to miR-22-inhibitor transfection, while the effect of miR-22-inhibitor was abrogated upon co-transfection with si-STK40 (*p* < 0.05). Taken together, miR-22 repressed the proliferation and differentiation of HFSCs by repressing STK40 and MEF2-ALP activity.

### EZH2 represses miR-22 to elevate STK40 thereby stimulating MEF2-ALP activity

**Figure 6:**
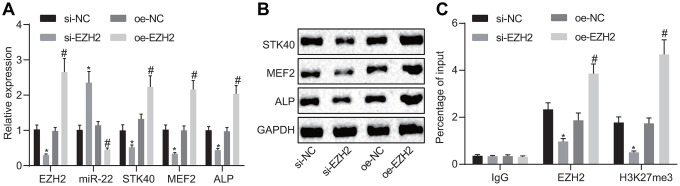
**EZH2 inhibits miR-22 expression to elevate STK40, which stimulates MEF2-ALP activity.** (**A**) The expression of miR-22, and mRNA expression of STK40, MEF2, ALP in HFSCs determined using RT-qPCR. (**B**) Protein expression of STK40, MEF2 and ALP in HFSCs normalized to GAPDH measured by Western blot analysis. (**C**) Enrichment of EZH2 or H3K27me3 in miR-22 promoter region evaluated using ChIP assay. * *p* < 0.05 *vs.* si-NC group; # *p* < 0.05 *vs.* oe-NC group; Measurement data were expressed as mean ± standard deviation. One-way ANOVA was utilized to compare data among multiple groups, followed by Tukey’s post hoc test. Cell experiments were conducted in triplicates.

EZH2 has been previously noted to regulate the expression of miR-22 by modulating its methylation [7]. The results of RT-qPCR (Figure 6A) demonstrated an elevated miR-22 expression, along with diminished mRNA expression of STK40, MEF2, and ALP in response to si-EZH2 transfection compared to transfection with si-NC, while reversed changes were evident in response to oe-EZH2 transfection in comparison to oe-NC (*p* < 0.05). Western blot analysis (Figure 6B) results illustrated reduced STK40, MEF2, and ALP protein expression in response to si-EZH2 transfection, but increased in response to oe-EZH2 transfection. Additionally, the ChIP assay (Figure 6C) was adopted to detect whether EZH2 and H3K27me3 were enriched in the miR-22 promoter region in HFSCs. Results demonstrated that enrichment of EZH2 or H3K27me3 in the miR-22 promoter region was evident upon treatment with oe-EZH2 compared to oe-NC (*p* < 0.05). On the basis of the aforementioned results, the conclusion could be drawn stating that elevation of EZH2 repressed miR-22 expression to upregulate STK40, which facilitated the MEF2-ALP activity.

### EZH2-mediated miR-22 suppression enhances HFSC proliferation and differentiation *in vitro* *via* MEF2-ALP axis

**Figure 7:**
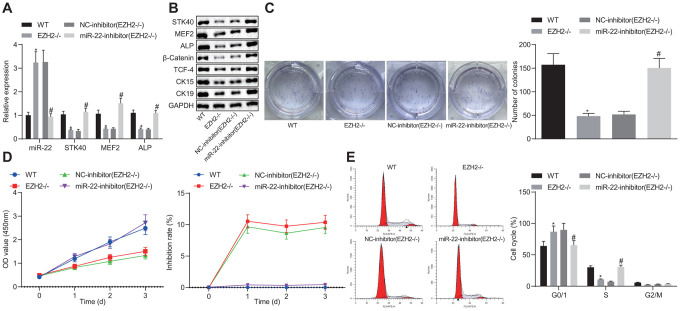
**EZH2-mediated miR-22 suppression promotes proliferation and differentiation of HFSCs.** (**A**) The expression of miR-22, and mRNA expression of STK40, MEF2 and ALP in HFSCs of EZH2^-/-^ mice or WT mice determined by RT-qPCR. (**B**) Protein expression of STK40, MEF2, ALP, differentiation-related proteins (β-catenin, TCF-4), and TA cell differentiation markers (CK15, CK19) in HFSCs of EZH2^-/-^ mice or WT mice normalized to GAPDH determined by Western blot analysis. (**C**) Colony forming capacity of HFSCs from EZH2^-/-^ mice or WT mice determined by colony formation assay. (**D**) The proliferation and viability from EZH2^-/-^ mice or WT mice evaluated by MTS assay. (**E**) HFSCs from EZH2^-/-^ mice or WT mice at different cell phases observed by flow cytometry. * *p* < 0.05 *vs.* WT mice; # *p* < 0.05 *vs.* NC-inhibitor (EZH2^-/-^) group; Measurement data were expressed as mean ± standard deviation. One-way ANOVA was utilized to compare data among multiple groups, followed by Tukey’s post hoc test. Repeated measures ANOVA was adopted to analyze data among multiple groups at different time points, followed by Bonferroni posttest. Cell experiments were conducted in triplicates.

To better understand the mechanism of EZH2-miR-22-STK40 axis on HFSC proliferation and differentiation, we extracted HFSCs in EZH2^-/-^ mice and characterized their expression pattern. RT-qPCR results (Figure 7A) revealed an evident increase in miR-22 expression along with diminished STK40, MEF2 and ALP mRNA expression in the HFSCs of EZH2^-/-^ mice. In HFSCs of EZH2^-/-^ mice transfected with the miR-22-inhibitor, STK40, MEF2 and ALP mRNA expression was elevated (*p* < 0.05). Further Western blot analysis (Figure 7B) results demonstrated that protein expression of STK40, MEF2, ALP, β-catenin, TCF-4, CK15 and CK19 was markedly reduced in the HFSCs of EZH2^-/-^ mice, while opposite results were induced by inhibition of miR-22 (*p* < 0.05). Colony formation assay and MTS results (Figure 7C, 7D) revealed markedly reduced colony forming and proliferation ability, along with increased inhibition rates of HFSCs from EZH2^-/-^ mice, while the effect of EZH2 knockout was abrogated upon inhibition of miR-22 (*p* < 0.05). Flow cytometric data (Figure 7E) revealed that more HFSCs were arrested in the G0/G1 phase in EZH2^-/-^ mice, while the effect of EZH2 knockout was abrogated upon inhibition of miR-22 (*p* < 0.05). Conjointly, down-regulating miR-22 by EZH2 could stimulate the proliferation and differentiation of HFSCs *via* MEF2-ALP axis *in vitro*.

### EZH2 knockout impairs HF keratinocyte differentiation and hair growth, but inhibits apoptosis *in vivo*

**Figure 8:**
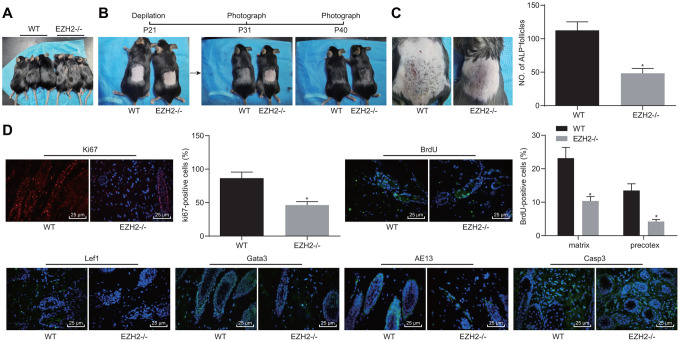
**EZH2 knockout inhibits HF keratinocyte differentiation and hair growth, but facilitated apoptosis *in vivo*.** (**A**) Hair loss exhibited by EZH2^-/-^ mice of 30 days old. (**B**) Delayed hair growth ability in EZH2^-/-^ mice. (**C**) HF in EZH2^-/-^ mice and WT mice determined by ALP staining. (**D**) Expression of proliferation, differentiation, and apoptosis markers EZH2^-/-^ mice and WT mice detected by immunofluorescence assay (400 ×). * *p* < 0.05 *vs.* WT mice; Measurement data were expressed as mean ± standard deviation. Unpaired *t* test was adopted to analyze the differences between two experimental groups if the data conformed to normal distribution and homogeneity of variance. n = 15.

The function of EZH2 on hair growth was further investigated on EZH2^-/-^ mice *in vivo*. As shown in Figure 8A, the hair loss was evident in EZH2^-/-^ mice. Delayed hair growth ability was also observed (Figure 8B). ALP staining results (Figure 8C) revealed the presence of a drastically lower proportion of HFs in EZH2^-/-^ mice than the WT mice (*p* < 0.05). In addition, immunofluorescence results (Figure 8D) demonstrated notable reductions in the numbers of cells positive for Ki67, BrdU, Lef1, Gata-3 and AE13, while the cleaved caspase 3 positive cells were potently increased, suggesting repressed HFSC proliferation, migration and keratinocyte differentiation, but increased HF keratinocyte apoptosis in EZH2^-/-^ mice (*p* < 0.05). The aforementioned results suggested that EZH2 knockout repressed HF keratinocyte differentiation and hair growth, but facilitated their apoptosis *in vivo*.

## DISCUSSION

**Figure 9:**
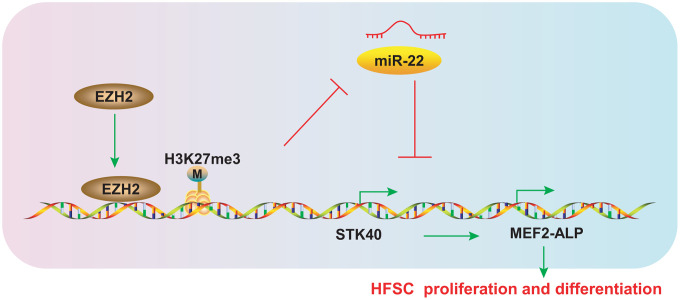
**Schematic diagram representing the role of the EZH2/miR-22/STK40/MEF2-ALP axis in HFSC proliferation and differentiation.** EZH2 inhibits miR-22 expression by accelerating H3K27me3 methylation, which in turn upregulates STK40, thereby accelerating proliferation and differentiation of HFSCs by activating MEF2-ALP activity.

HFs are progressively miniaturized by HFSC aging, which eventually leads to hair loss [15]. Early HFSCs can maintain the stemness and slow-cycling abilities during the differentiation process into TA cells, which is crucial for the next hair cycle [16]. The epigenetics field represents the potential for the discovery of new molecular biomarkers so as to prevent or alleviate hair loss, and uncovering the mechanisms underlying hair growth. The evidence from our study validates the hypothesis that EZH2-mediated miR-22 down-regulation elevated the expression of STK40 to facilitate MEF2-ALP activity, thereby stimulating HFSC differentiation and hair growth (Figure 9).

Initially, an elevated STK40 expression was observed during HFSC differentiation. In consistency with our study, an existing study mentioned that STK40 down-regulation lowered the expression of hair growth and hair differentiation markers [10]. Moreover, a prior study also reported that STK40 was implicated in the viability of keratinocytes [17]. In addition, we found that downregulation of STK40 suppressed the differentiation, but facilitated the apoptosis of HFSCs. Our *in vivo* experiments conducted in STK40^-/-^ mice further confirmed these results, as substantiated by the diminished number of cells positive for Ki67, BrdU, Lef1, Gata-3, and AE13, with an increase in the proportion of cleaved caspase 3-positive cells. Ki67 was considered as a viable marker for cell proliferation, and BrdU staining has been adopted for the identification of surviving and proliferating cells [18]. Transcription factor Lef1 serves as an essential biomarker for HF-derived neural crest stem cells melanocytic differentiation [19]. GATA3 contributed to the differentiation and survival of parathyroid progenitor cells [20]. Moreover, AE13 facilitated the differentiation of all epithelial lineages of the HF [21]. However, an increased level of cleaved caspase-3 was indicative of diminished cell viability and a marker for cell apoptosis [22]. The *in vivo* experimentation also suggested that STK40 knockout repressed hair growth.

Furthermore, STK40 evidently demonstrated ability to facilitate MEF2-ALP activity so as to stimulate the proliferation and differentiation of HFSCs. MEF2 was identified as a target of STK40 *via* histone deacetylase 5 [11], which subsequently elevated the expression of ALP [13]. Meanwhile, ALP functioned as an essential marker to promote hair growth [14]. β-catenin has been regarded as an imperative marker for differentiation [23]. The downregulated TCF-4 was reported to comprehensively inhibit the proliferative and invasive ability of lung cancer cells [24]. In addition, CK15 and CK19 belonged to the class of HFSC markers [25]. In the current study, diminished expression patterns of β-catenin, TCF-4, CK15 and CK19 were evident in response to either STK40 knockout or MEF2 silencing. The regulatory effects of STK40 knockout could be reverted by si-MEF2, indicating that STK40 down-regulation impaired the proliferation and differentiation of HFSCs *via* MEF2-ALP axis.

Fundamentally, miR-22 could negatively regulate STK40 expression, thereby modulating MEF2-ALP activity and its down-regulation resulted in facilitated HFSC differentiation and hair growth. Similarly, down-regulation of miR-22 contributed to hair growth since it delayed access to catagen and intrinsically expedited the transition from telogen to anagen [6]. In the current study, diminished expression of β-catenin, TCF-4, CK15 and CK19 was evident upon miR-22 inhibition. In addition, we also found that miR-22 was repressed by EZH2, thereby enhancing the proliferation and differentiation of HFSCs. A prior study documented the ability of EZH2 to repress miR-22 in hepatocellular carcinoma [7]. Notably, the absence of EZH2 was reported to be defective in proliferation of HFs [26]. We also observed diminished expression of β-catenin, TCF-4, CK15 and CK19 in the HFSCs of EZH2^-/-^ mice and the loss of EZH2 suppressed the differentiation, but facilitated the apoptosis of HFSCs. Our *in vivo* experiments conducted in EZH2^-/-^ mice also verified our finding, which displayed that the number of cells positive for Ki67, BrdU, Lef1, Gata-3 and AE13 were all markedly reduced, while cleaved caspase 3-positive cells were potently increased.

In conclusion, the current study sheds light on the underlying mechanism for the proliferation and differentiation of HFSCs and hair growth. Specifically, EZH2, which was upregulated in HFSCs, repressed miR-22 by accelerating H3K27me3 methylation, thereby elevating STK40 to facilitate MEF2-ALP activation. The aforementioned mechanism might be responsible for HFSC differentiation and hair growth, which proposed EZH2 and EZH2-mediated inhibition of miR-22 as promising targets for preventing or alleviating hair loss in the future. However, attention should be paid to the side effect on immune system and clinical effect related to malignancies due to the interplay between EZH2 and other miRNAs implicated in the drug resistance and tumor progression [9, 27, 28]. Since STK40 is elevated upon miR-22 downregulation, drugs related to STK40 promotion may reduce the incidence of clinical side effects. Additionally, encouraging as findings presented in the current investigation, miRNA-based therapeutic approaches remain in their infancy in clinical application. Therefore, more detailed studies should be conducted for further exploration in the clinical setting.

## MATERIALS AND METHODS

### Experimental animals

Forty-five C57BL/6 mice (3 weeks old, 15 - 21 g) were acquired from the Experimental Animal Center of Zhengzhou University, including 15 EZH2 knockout (^-/-^) mice, 15 wild type (WT) mice and 15 STK40^-/-^ mice respectively. All animals were housed in specific pathogen free facilities.

### Isolation and characterization of HFSCs

WT mice, EZH2^-/-^ or STK40^-/-^ (9 days postnatal) were anesthetized and the hair on the back was shaved with electric scissors to avoid mutilation to the skin and subcutaneous tissues. Then, 70% ethanol was applied for disinfection and removal of the remaining hair residues. The entire skin was dissected and immersed in trypsin (GIBCO, Carlsbad, CA, USA) with the dermis facing down at 4°C O/N. Single-cell suspension was subsequently prepared by dissociating the epidermis and HF from the dermis. The cells were rinsed using phosphate buffer saline (PBS) containing 5% fetal bovine serum (FBS), and filtered using a 70 μm and then 40 μm cell strainer, respectively. The cell suspension was incubated with the experimental antibody for 90 min on ice. The antibodies used were as follows: Alpha6-PE (1 : 500; eBioscience, San Diego, CA, USA) and CD34-eFluor660 (1 : 100, eBioscience). Dead cells were eliminated using 4', 6-diamidino-2-phenylindole (DAPI). MoFlo XDP sorters (Beckman Coulter Inc., Brea, CA, USA) equipped with the Summit 5.2 software were adopted for definitive cell isolation [6].

### Cell culture and transfection

HFSC differentiation to TA cells was induced by overexpressing β-catenin and c-myc (GeneChem, Shanghai, China). The medium was renewed every 3 days. Cell growth, proliferation and differentiation were observed under an inverted microscope (Ti-E, Nikon, Tokyo, Japan) [3]. HFSCs (200 μL/well) in the logarithmic growth phase were seeded onto a 6-well plate and placed in antibiotic-free complete medium. Upon reaching 30% - 50% confluence, the HFSCs were transfected strictly under the protocols of Lipofectamine 2000 (Invitrogen, Carlsbad, CA, USA). Transfected HFSCs were incubated at 37°C (5% CO_2_) for 6 - 8 h. After complete medium renewal, the HFSCs were finally incubated for 24 - 48 h at 37°C for subsequent experiments. HFSCs from the WT mice were transfected with the negative control (NC)-mimic, miR-22-mimic, NC-inhibitor, miR-22-inhibitor, miR-22-inhibitor + siRNA (si)-NC, miR-22-inhibitor + si-STK40, si-NC, si-EZH2, oe-NC, oe-EZH2, si-NC, or si-MEF2. HFSCs from the EZH2^-/-^ mice were treated with the NC-inhibitor (EZH2^-/-^) or miR-22-inhibitor (EZH2^-/-^).

### Flow cytometry

Subsequently, 48-h post transfection, the cells were collected and centrifuged, with elimination of the supernatant. The cells were re-suspended using PBS and the cell concentration was adjusted to 1 × 10^5^ cells/mL. The cells were fixed using 75% ethanol for 1 h and then subjected to centrifugation with removal of the ethanol. The cells were incubated with 100 μL of RNase A in a 37°C water bath devoid of light and then 400 μL propidium iodide (Sigma, St Louis, MO, USA) was supplemented, followed by 30-min incubation at 4°C in conditions devoid of light. Flow cytometry was adopted to monitor the cell cycle progression by detection of red fluorescence at an excitation wavelength of 488 nm.

### Colony formation assay

Transfected cells (500 μL/well) were seeded onto a 6-well plate and cultured overnight. Mitomycin (5 μg/mL) was applied to treat the cells for 24 h the following day, with mitomycin-free complete medium renewal. Fourteen days later, the cells were fixed using 4% paraformaldehyde, stained using 0.1% crystal violet (20 min), and then counted under the microscope. The experiment was repeated 3 times in triplicates.

### MTS cell proliferation assay

MTS assay was conducted to determine cell proliferation in strict accordance with the manufacturer’s instruction (Promega, Madison, WI, USA). Briefly, the cells were seeded onto 96-well plates, and cultured for 24 h at 37°C before transfection. Then, the cells were transfected with the corresponding oligonucleotides. MTS solution (20 μL) was supplemented into each well at different time points, followed by culture for 1 h at 37°C. Absorbance was measured at an excitation wavelength of 450 nm.

### Whole-mount HF neogenesis assay

HF neogenesis assay was conducted as previously described [6]. Briefly, the mice were anesthetized using pentobarbital. A full-thick excisional wound (1 cm^2^ full thickness) was created at the mid back of 3-week old mice. The skin was immersed in ethylenediaminetetraacetic acid (EDTA)-PBS overnight (37°C) to determine the extent of newly-grown HFs in the wound. The epidermis was gently peeled off under a dissecting microscope, and fixed using 4% paraformaldehyde for 1 h, followed by blockage using 3% H_2_O_2_. ALP immunostaining was then conducted in 1.5 mL Eppendorf tubes. The dermis was fixed using acetone overnight (4°C) and then incubated with the nitro blue tetrazolium chloride/5-Bromo-4-chloro-3-indolyl phosphate substrate solution (Roche, Basel, Switzerland). EDTA (20 mM) was added into the PBS solution to terminate the reaction.

### Immunofluorescence assay

Immunofluorescence was performed as described previously [29]. Briefly, the skin samples were isolated from mice (30 days), and fixed using 4% paraformaldehyde, followed by embedding in paraffin and dissection into 5-μm sections. Paraffin-embedded sections were microwave pretreated, and incubated with corresponding primary and secondary antibodies (Invitrogen), followed by DAPI staining. The corresponding antibodies were as follows: bromodeoxyuridine (BrdU; ab8152, mouse, 1 : 200, Abcam, Cambridge, UK), cleaved caspase-3 (ab13847, rabbit, 1 : 100, Abcam), GATA binding protein 3 (Gata-3; #5852, rabbit, 1 : 1600, Cell Signaling Technology, Beverly, MA, USA), lymphoid enhancer factor1 (Lef1; #2230, rabbit, 1 : 200, Cell Signaling Technology), Ki67 (#12075, rabbit, 1 : 50, Cell Signaling Technology), and alpha-esterase 13 (AE13; ab16113, mouse, 1 : 100, Abcam).

Immunofluorescence for HFSCs: HFSCs were placed on poly-d-lysine-coated coverslips, fixed using 4% paraformaldehyde for 20 min, and then permeabilized using 0.1% Triton X-100/PBS for 3 min. The HFSCs were then incubated with the following specific primary antibodies: Lef1 (#2230, rabbit, 1 : 200, Cell Signaling Technology), K19 (#12434, rabbit, 1 : 50, Cell Signaling Technology), CD200 (sc-71762, mouse, 1 : 100, Santa Cruz, Santa Cruz, CA, USA), and β1-integrin (ab95623, Rat, 1 : 500, Abcam).

### Dual-luciferase reporter gene assay

The 293T cells (2 × 10^5^ cells/well) in different groups were seeded onto 6-well plates. Upon cell adherence, the cells were transfected for 48 h. After transfection, the cells were collected and the luciferase activities of miR-22 and STK40 were analyzed strictly under the protocols of the dual-luciferase reporter kit provided by Genecopoeia (D0010, purchased in Solarbio, Beijing, China). Glomax20/20 luminometer provided by Promega (E5311; purchased from Shaanxi Zhongmei Biotechnology Co., Ltd., Shanxi, China) was adopted to assess the luminance.

### Chromatin immunoprecipitation (ChIP)

EZ-Magna ChIP kit (EMD Millipore, Bedford, MA, USA) was adopted to perform ChIP assay. The HK2 cells were fixed using 4% paraformaldehyde and incubated with glycine for 10 min to facilitate the formation of the DNA-protein crosslink. Cell lysis buffer and nuclear lysis buffer were added to lyse cells after which chromatin fragmentation (200 - 300bp) was generated by sonication. Magnetic beads coupled to Protein A containing antibodies were then supplemented to the immuno-precipitate lysate. Cells in the NC group, anti-EZH2 group and anti-H3K27 group were incubated with respective antibodies to immunoglobulin G (IgG) (ab171870, Abcam), EZH2 (#5246, 1 : 100, Rabbit, Cell Signaling Technology), and trimethylated histone H3 at lysine 27 (H3K27me3) (#9733, 1 : 50, Rabbit, Cell Signaling Technology) respectively. RT-qPCR was conducted to analyze the expression of the precipitated DNA.

### RT-qPCR

Total RNA was extracted using TRIzol (Invitrogen). EZH2, miR-22, STK40, MEF2 and ALP primers were synthesized by Invitrogen and their sequences are listed in Table 1. The extracted RNA was reverse-transcribed into cDNA using the TaqMan™ MicroRNA Reverse Transcription Kit (4366596; Thermo Fisher Scientific, NY, USA) or High-Capacity cDNA Reverse Transcription Kit (4368813; Thermo Fisher Scientific). Real-time qPCR was then conducted using the SYBR^®^Premix Ex TaqTMII kit (Tli RNaseH Plus; RR820A, TaKaRa, Tokyo, Japan) on the ABI7500 instrument (Thermo Fisher Scientific) with U6 and glyceraldehyde-3-phosphate dehydrogenase (GAPDH) serving as internal references. Reaction solution of PCR was placed on real-time fluorescent qPCR (ABI, Foster City, CA, USA) for PCR. The fold changes were calculated by relative quantification (2^-ΔΔCt^ method).

**Table 1:** Primer sequences for RT-qPCR.

**Target Gene**	**Forward (5' - 3')**	**Reverse (5' - 3')**
EZH2	AGGACGGCTCCTCTAACCAT	CTTGGTGTTGCACTGTGCTT
miR-22	GGGGGATCCCTGGGGCAGGACCCT	GGGGAATTCAACGTATCATCCACCC
MEF2	GGCTTTGTCCAGCTCCACT	ATCCCGATGCAGACGATTCAG
ALP	GTTGCCAAGCTGGGAAGAACAC	CCCACCCCGCTATTCCAAAC
STK40	GCAAGGAATAGAGAGCCAAG	TACCATCCGACCAGACTCTG
U6	GCTTCGGCAGCACATATACTAAAAT	CGCTTCACGAATTTGCGTGTCAT
GAPDH	GCACAGTCAAGGCCGAAAT	GCCTTCTCCAATGGTGGTGAA

Note: RT-qPCR, reverse transcription quantitative polymerase chain reaction; EZH2, enhancer of zeste homolog 2; miR-22, microRNA-22; MEF2, myocyte enhancer factor 2; ALP, alkaline phosphatase; STK40, serine/threonine kinase 40; GAPDH, glyceraldehyde-3-phosphate dehydrogenase.

### Western blot analysis

Radioimmunoprecipitation assay kit (R0010; Solarbio) was adopted to extract the total protein from the HFSCs in skin tissues or from different transfection groups. Bicinchoninic acid protein assay kit (GBCBIO Technologies; Guangzhou, Guangdong, China) was employed to detect the protein concentration. Proteins were separated using 10% electrophoresis and then transferred onto polyvinylidene fluoride membranes. After being blocked in Tris-Buffered Saline Tween-20 solution containing 5% bovine serum albumin at ambient temperature, the membrane was probed with the rabbit antibodies to GAPDH (#5174, 1 : 1000, Cell Signaling Technology), EZH2 (#5246, 1 : 1000, Cell Signaling Technology), STK40 (1 : 100, ab135747, Abcam), MEF2 (#5030, 1 : 1000, Cell Signaling Technology), and ALP (ab83259, 1 : 1000, Abcam), followed by incubation at 4°C on a shaking table. Afterwards, the goat anti-rabbit antibody to IgG (ab150077, 1 : 1000, Abcam) was added and incubated with the membrane at ambient temperature. The membrane was developed using enhanced chemiluminescence and the grey value of bands was analyzed using the ImageJ software. Relative protein expression was analyzed as the ratio of gray value of protein band to be tested to that of the internal reference.

### Statistical analysis

All data were processed and analyzed using the SPSS 21.0 statistical software (IBM Corp., Armonk, NY, USA). Measurement data were expressed as mean ± standard deviation. If the data conformed to normal distribution and homogeneity of variance, unpaired *t* test was adopted to analyze the differences between two experimental groups, while one-way analysis of variance (ANOVA) was utilized to compare data among multiple groups, followed by Tukey’s post hoc test. Repeated measures ANOVA was adopted to analyze data among multiple groups at different time points, followed by Bonferroni posttest. A value of *p* < 0.05 was considered to be statistically significant.

### Ethical statement

The study was conducted with approval of the Animal Ethics Committee of the First Affiliated Hospital of Zhengzhou University, and in strict accordance with the recommendations of the Guide for the Care and Use of Laboratory animals published by the National Institutes of Health.
